# Pre-operative systemic inflammatory response index influences long-term survival rate in off-pump surgical revascularization

**DOI:** 10.1371/journal.pone.0276138

**Published:** 2022-12-15

**Authors:** Tomasz Urbanowicz, Anna Olasińska-Wiśniewska, Michał Michalak, Bartłomiej Perek, Ahmed Al-Imam, Michał Rodzki, Anna Witkowska, Ewa Straburzyńska-Migaj, Michał Bociański, Marcin Misterski, Maciej Lesiak, Marek Jemielity

**Affiliations:** 1 Cardiac Surgery and Transplantology Department, Poznan University of Medical Sciences, Poznan, Poland; 2 Department of Computer Science and Statistics, Poznan University of Medical Sciences, Poznan, Poland; 3 Department of Anatomy and Cellular Biology, College of Medicine, University of Baghdad, Baghdad, Iraq; 4 1^st^ Cardiology Department, Poznan University of Medical Sciences, Poznan, Poland; Royal Infirmary of Edinburgh, UNITED KINGDOM

## Abstract

Coronary artery bypass revascularization is still the optimal treatment for complex coronary artery disease with good long-term results. The relation between inflammatory activation in the post-operative period and the long-term prognosis was already postulated. The possible predictive role of preoperative inflammatory indexes after the off-pump coronary artery bypass grafting technique on long term survival was the aim of the study. Study population included 171 patients with a median age of 64 years (59–64) operated on using off-pump technique between January and December 2014. Patients enrolled in the current study were followed-up for 8 years. We conducted a multivariable analysis of pre-operative and post-operative inflammatory markers based on analysis of the whole blood count. The overall survival rate was 80% for the total follow-up period, while 34 deaths were reported (30-days mortality rate of 1%). In the multivariable analysis, a pre-operative value of systemic inflammatory response index (SIRI) >1.27 (HR = 6.16, 95% CI 2.17–17.48, p = 0.012) revealed a prognostic value for long-term mortality assessment after off-pump surgery. Preoperative inflammatory activation evaluated by systemic inflammatory reaction index (SIRI) possess a prognostic value for patients with complex coronary artery disease. The SIRI value above 1.27 indicates a worse late prognosis after off-pump coronary artery bypass (AUC = 0.682, p<0.001).

## Introduction

Diffuse atherosclerotic disease of the coronary arteries limits the daily activity and life span of patients due to insufficient blood supply of the myocardium. Concerning long-term results, coronary artery bypass revascularization is still the optimal treatment for complex coronary artery disease management [1]. The surgical revascularization allows for the complex provision of coronary blood supply to the heart due to multiple grafts utility [2]. The arterial grafts provide superior results to venous ones which are commonly applied in clinical practice [3].

Coronary artery disease evolves from a chronic inflammatory process that leads to atherosclerotic changes influencing the survival rate [4–6]. The atherosclerotic plaques initiation and progression depend on an imbalance between pro-inflammatory and anti-inflammatory changes in the endothelium after triggering the inflammation reaction chain [7, 8].

The surgical revascularization can be performed with the utility of cardiopulmonary bypass (CPB) or with the off-pump technique [9, 10]. The omittance of CBP results in diminishing of the systemic inflammatory reaction which is the critical factor for long-term mortality risk [11]. The relation between inflammatory activation in the peri-operative period and the long-term prognosis was already postulated [8, 12].

Researchers proved the significant role of inflammatory indices in clinical practice in the prediction of the post-operative morbidity and mortality [13, 14]. The postulated indices include systemic inflammatory index (SII), systemic inflammatory response index (SIRI), and aggregate inflammatory systemic index (AISI). The clinical significance of post-operative indices in surgical coronary artery revascularization in mortality prediction has been already presented [15, 16]. Little is however known on the significance of the inflammatory indices on the long-term mortality after off-pump coronary bypass surgery.

We present the results of a single-center retrospective analysis of patients’ survival after surgical revascularizations with the off-pump technique. The study aimed to evaluate pre-operative inflammatory markers for long-term outcomes prediction.

## Material and methods

**Fig 1:**
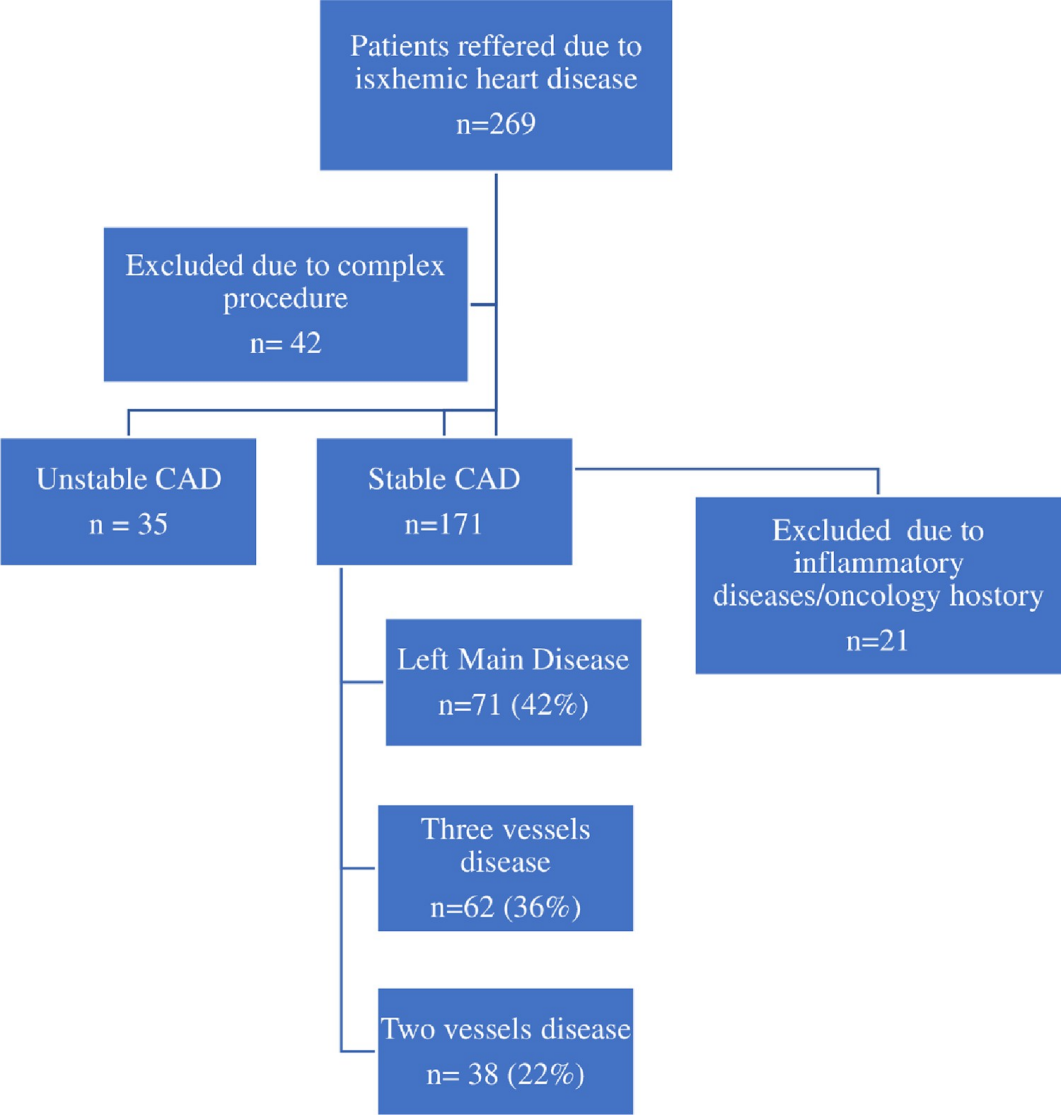
Inclusion criteria for the study.

The current study included 171 patients operated on in our Department between January and December 2014 as presented in Fig 1.

There were 71 (42%) patients with left main disease, 62 (36%) and 38 (22%) with diagnosis of three and two vessels disease, respectively. The indication for surgery was a chronic coronary syndrome. All patients were operated on by the experienced surgical team with the use the off-pump technique, and then followed up for a period of eight years. The exclusion criteria were concomitant procedures and haematological or oncological diseases. Moreover, patients with acute coronary syndromes were not taken into consideration.

Demographic and clinical data were collected. Although we focused in our study on inflammatory parameters obtained from whole blood count, the analysis was performed including additional laboratory parameters (troponin-I serum level, serum creatinine concentration, LDL cholesterol serum fraction) to obtain broader spectrum and distinguish significant factors. The history of previous peripheral vascular disease and events (PVE), including stroke and peripheral arterial disease (PAD) was analysed.

Pre-operative and post-operative (24 hours) whole blood results were obtained, including neutrophils, lymphocytes and platelets counts. Troponin-I levels were recorded after 4, 24 and 48 hours after surgery. The analysed inflammatory indexes were calculated as follows—systemic inflammatory index (SII)—quotient of neutrophils and platelets divided over lymphocyte counts; systemic inflammatory response index (SIRI)—quotient of neutrophil and monocyte divided by lymphocyte counts, and aggregate inflammatory systemic index (AISI)—quotient of neutrophils, monocytes and platelets divided by lymphocytes count.

C-reactive protein was not routinely measured as patients with infection suspicion were ruled out from the study. Postoperatively, C- reactive protein was not performed routinely except of patients presenting infection symptoms.

Echocardiography was performed in all patients before and after the surgery, and at the discharge.

The surgical team operated on all patients under general anaesthesia with the off-pump technique through median sternotomy. The anastomoses were performed using intraluminal shunt application as the target coronary artery segment was stabilized by Octopus (Medtronic, USA). The anastomosis was performed with a single running 7–0 monofilament suture.

The mortality analysis was based on all-cause mortality, and the researchers confirmed the information concerning deaths in the National Healthcare Database.

The study was approved by the Institutional Ethics Committee (No 914/21 and date of approval: 24 November 2021) and respected the principles outlined in the Declaration of Helsinki.

### Statistical analysis

All continuous data were presented as medians and interquartile ranges Me (Q_1_-Q_3_) for non-normal distribution. A non-parametric test (Mann-Whitney) was used to compare the continuous variables. A receiver characteristic curve analysis was performed to reveal potential predictors for mortality. A proportional hazard regression model was used to assess mortality risk factors. Both univariable and multivariable analyses (stepwise backward selection) were performed. The results were presented as hazard ratios (HR) and 95% confidence intervals (95% CI). The statistical analysis was performed using MedCalc statistical package; MedCalc® Statistical Software version 20.010 (MedCalc Software Ltd, Ostend, Belgium; https://www.medcalc.org; 2021). All tests were considered significant at p<0.05.

## Results

The study group comprised 171 patients (152 (89%) males and 19 (11%) females) with a median age of 64 (59–64). The overall survival rate was 80% during the follow-up period, while 34 deaths were reported. The 30 days mortality rate was 1% (2 patients), and the mean hospitalization time was 11 +/- 2 days.

The study patients suffered from several co-morbidities, including arterial hypertension (152 (89%)), hypercholesterolemia (98 (57%)), diabetes mellitus (DM) (65 (38%)), peripheral arterial disease (PAD) (29 (17%)), history of stroke (19 (11%)) and chronic obstructive pulmonary disease (COPD) (16 (9%)). Preoperatively, the mean left ventricular diastolic diameter and left ventricular ejection fraction were 48mm (44–52) and 55% (50–60), respectively. There was no difference between the survivors and deaths group regarding the pre-operative clinical data except for hypercholesterolemia (p = 0.034) though the LDL cholesterol serum fraction was insignificant (p = 0.875), as presented in the Table 1.

**Table 1:** Characteristics of survivors and non-survivors’ groups.

Pre-operative parameters	Survivors (n = 137)	Non-survivors (n = 34)	p-value
Clinical characteristics:			
1. Age (years) median(Q1-Q3)	63 (59–67)	66 (63–73)	0.020
2. Gender (M (%)/F (%))	121 (88%) / 16 (12%)	31(91%) / 3 (9%)	0.635
Co-morbidities:			
1. Arterial hypertension	120 (88%)	32 (94%)	0.278
2. Hypercholesterolemia	84 (61%)	14 (41%)	0.034*
3. COPD	10 (7%)	6 (18%)	0.637
4. History of stroke	12 (9%)	7 (21%)	0.495
5. Peripheral artery disease	20 (15%)	9 (27%)	0.099
6. Diabetes mellitus	50 (37%)	15 (44%)	0.413

Abbreviations: AISI- aggregate inflammatory systemic index, F–female, M–male, n–number, SII–systemic inflammatory index, SIRI–systemic inflammatory response index, Q-quartile, WBC–white blood cell count. *—statistical significance.

The mean number of performed grafts was 2.2 +/- 0.1 and 2.2 +/- 0.2 in survivors and deaths groups, respectively.

**Fig 2:**
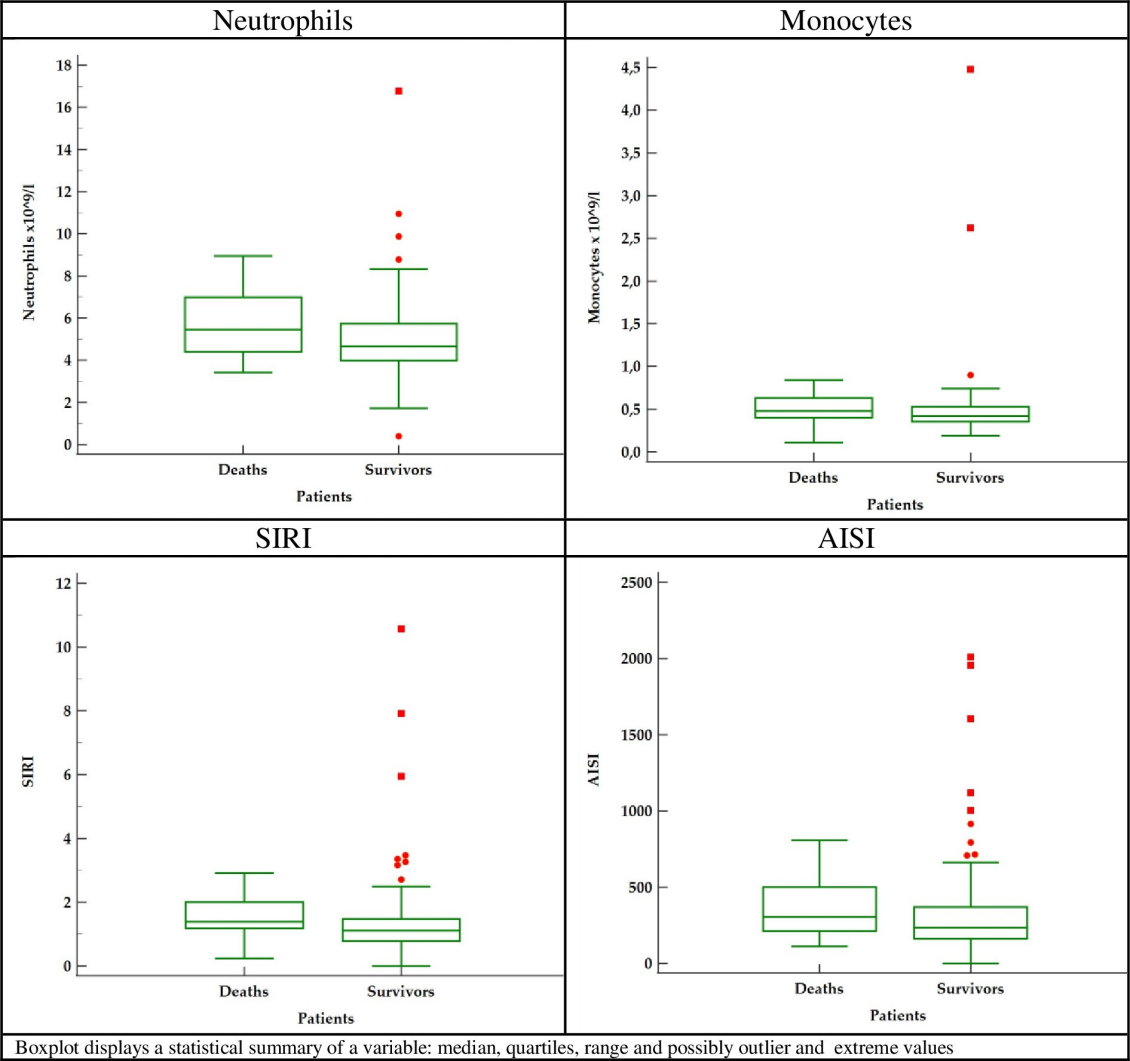
Box-plot 1. Preoperative significant parameters.

Maximum post-operative Troponin-I were 1.97 (0.68–3.97) ng/ml in survivors vs 1.86 (0.46–4.92) in deaths subgroups (p = 0.667), respectively. We retrieved the complete blood count test results before surgery and 24 hours after (Table 2) and presented the significant preoperative parameters in Fig 2.

**Table 2:** Comparison of pre-operative and post-operative results between survivors and non-survivors groups.

Pre-operative parameters	Survivors (n = 137)	Non-survivors (n = 34)	p-value
Clinical characteristics:			
1. Age (years) median(Q1-Q3)	63 (59–67)	66 (63–73)	0.020
2. Gender M/F (%)	121 (88%) / 16 (12%)	31(91%) / 3 (9%)	0.635
Preoperative laboratory results:			
1. WBC x10^9^/l (median(Q1-Q3))	7.47 (6.51–8.33)	7.94 (7.55–9.88)	0.058
2. Lymphocytes x10^9^/l (median(Q1-Q3))	1.83 (1.47–2.27)	1.83 (1.57–2.05)	0.976
3. Neutrophils x10^9^/l (median(Q1-Q3))	4.66 (4.02–5.73)	5.45 (4.40–6.99)	0.009*
4. Hemoglobin mmol/l (median(Q1-Q3))	8.90 (8.20–9.45)	8.55 (8.00–9.10)	0.133
5. Platelets x10^3^/l (median(Q1-Q3))	213 (188–259)	220 (180–272)	0.843
6. Monocytes x10^9^/l (median(Q1-Q3))	0.42 (0.35–0.53)	0.48 (0.40–0.63)	0.021
7. SIRI (median(Q1-Q3))	1.11 (0.78–1.47)	1.39 (1.18–2.01)	<0.001*
8. SII (median(Q1-Q3))	562 (409–792)	618 (498–1017)	0.106
9. AISI (median(Q1-Q3))	235 (162–369)	305 (213–500)	0.031*
10. Serum creatinine umol/L (median(Q1-Q3))	97 (81–121)	99 (79–124)	0.745
11. Troponin–I ng/mL (median(Q1-Q3))	0.02 (0.01–0.03)	0.02 (0.01–0.03)	0.901
12. LDL serum level mg/dL (median(Q1-Q3))	58 (51–66)	60 (52–69)	0.875
Number of grafts	2.2 +/- 0.1	2.2 +/- 0.2	0.897
Postoperative laboratory results:			
1. WBC x10^9^/l (median(Q1-Q3))	8.51 (7.125–10.01)	8.51 (7.13–10.01)	0.955
2. Lymphocytes x10^9^/l (median(Q1-Q3))	1.85 (1.53–2.34)	1.77 (1.33–2.23)	0.151
3. Neutrophils x10^9^/l (median(Q1-Q3))	5.53 (4.03–6.55)	5.53 (4.13–7.08)	0.447
4. Hemoglobin mmol/l (median(Q1-Q3))	7.06 (6.6–7.55)	6.75 (6.50–7.40)	0.233
5. Platelets x10^3^/l (median(Q1-Q3))	309 (244–355)	255 (199–317)	0.041*
6. Monocytes x10^9^/l (median(Q1-Q3))	1.02 (0.73–1.17)	0.88 (0.64–1.18)	0.446
7. SIRI (median(Q1-Q3))	2.46 (1.62–3.55)	2.60 (1.73–5.51)	0.329
8. SII (median(Q1-Q3))	764 (512–1109)	864 (597–1059)	0.633
9. AISI (median(Q1-Q3))	692 (424–1232)	651 (484–1425)	0.998

Abbreviations: AISI—aggregate inflammatory systemic index, F–female, M–male, n–number, SII–systemic inflammatory index, SIRI–systemic inflammatory response index, Q-quartile, WBC–white blood cell count. *—statistical significance.

**Fig 3:**
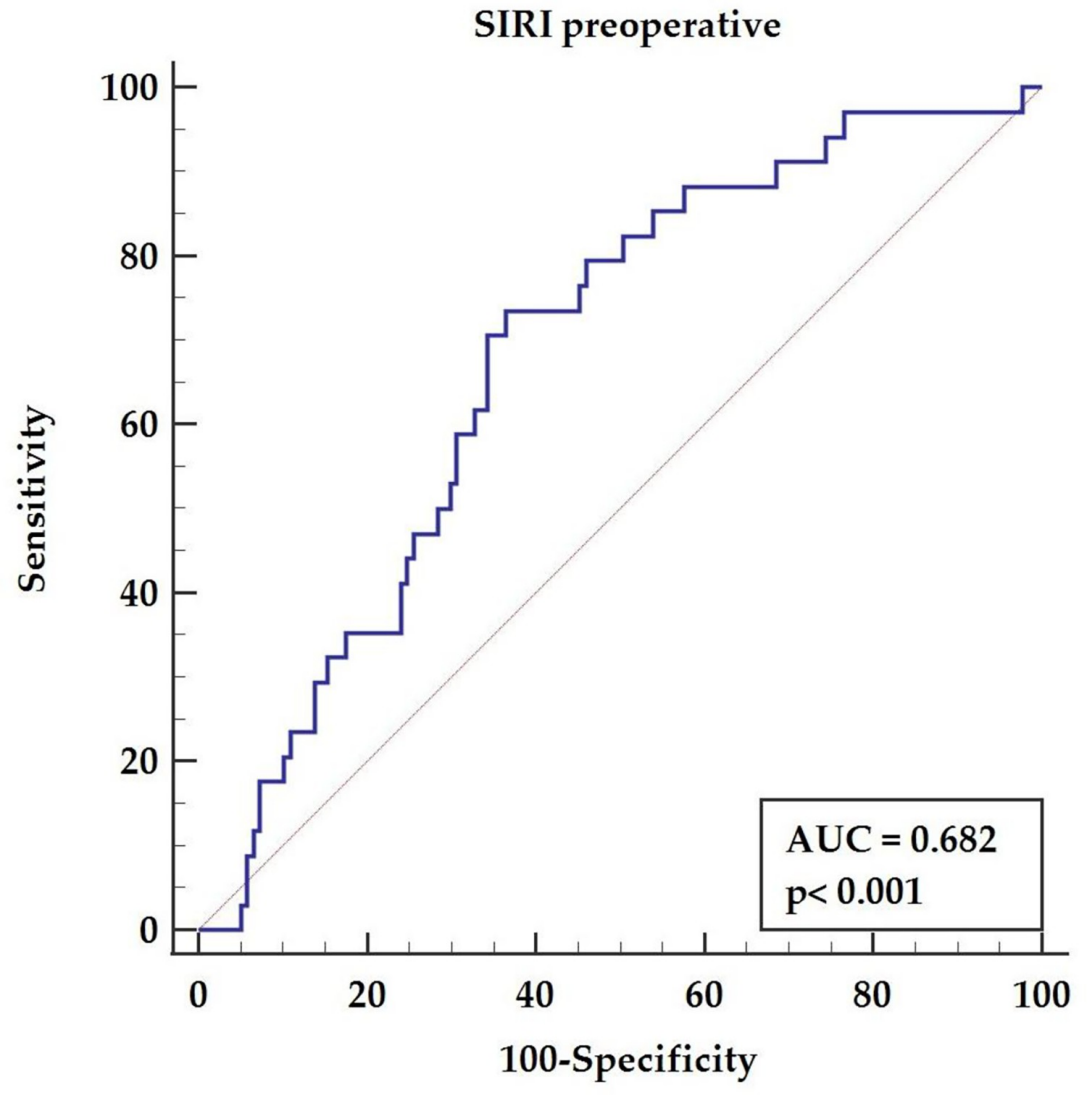
The receiver-operating characteristic curve for the pre-operative value of systemic inflammatory response index.

The receiver-operating characteristic (ROC) curve analysis confirmed a significant effect of the pre-operative SIRI (AUC = 0.682, p<0.001) with a sensitivity of 73.53% and specificity of 63.5%, and a cut-off value above 1.27, as presented in Fig 3.

### Univariable analysis

According to the univariate Cox regression analysis (Table 3), significant preoperative factors included PVE (summed stroke and peripheral artery disease) (HR = 2.70, 95% CI 1.17–6.26, p = 0.020), left ventricle diastolic diameter above 45mm (HR = 6.09, 95% CI 1.46–25.48, p = 0.013) and left ventricle ejection fraction below 50% (HR = 2.72, 95% CI 1.36–5.22, p = 0.005).

**Table 3:** Univariable analysis results.

Parameters	HR	95% CI	p-value
Stroke	2.70	1.17–6.26	0.020*
Pre-operative echocardiographic results:			
1. LVd	1.07	1.02–1.12	0.009*
2. LVd > 45 mm	6.09	1.46–25.48	0.013*
3. LVEF	0.95	0.92–0.98	0.001*
4. LVEF < 50%	2.72	1.36–5.44	0.005*
Pre-operative laboratory results:			
1. Neutrophils above 5.2	2.59	1.29–5.22	0.007*
2. Monocytes > 0.44	2.83	2.31–6.13	0.008*
3. SIRI > 1.27	1.39	1.72–7.69	0.001*
4. AISI >362	2.28	1.13–4.76	0.020*
Postoperative echocardiographic results:			
1. LVd > 48 mm	4.06	1.67–9.85	0.002*
2. LVEF < 50%	2.56	1.28–5.13	0.008*

Abbreviations: AISI—aggregate inflammatory systemic index, LVd–left ventricle diameter, LVEF–left ventricle ejection fraction, SIRI–systemic inflammatory response index.

Significant preoperative laboratory markers for long-term prognosis prediction included—the neutrophils count above 5.2 (HR = 2.59, 95% CI 1.29–5.22, p = 0.007), monocytes count above 0.44 (HR = 2.83, 95% CI 6.13–11.39, p = 0.008), SIRI above 1.27 (HR = 1.39, 95% CI 1.72–7.69, p = 0.001) and AISI > 362 (HR = 2.28, 95% CI 1.13–4.76, p = 0.020). The postoperative echocardiographic results significant for mortality prediction included the left ventricle diastolic diameter above 48 mm (HR = 4.06, 95% CI 1.67–9.85, p = 0.002) and ejection fraction below 50% (HR = 2.56, 95% CI 1.28–5.13, p = 0.008).

### Multivariable analysis

We deployed multivariate analysis for parameters presenting as significant in univariable analysis, and novel data emerged concerning the significance concerning age (HR = 1.09, 95% CI 1.03–1.16, p = 0.005), COPD (HR = 5.24, 95% CI 1.84–14.91, p = 0.002), stroke (HR = 3.29, 95% CI 1.31–8.29, p = 0.012), and the preoperative left ventricle ejection fraction (HR = 3.28, 95% CI 1.50–7.16, p = 0.003). Significant pre-operative laboratory markers obtained from whole blood count included SIRI above 1.27 (HR = 6.16, 95% CI 2.17–17.48, p = 0.001) as presented in Table 4.

**Table 4:** Multivariable analysis results.

Parameters	HZ	95% CI	p-value
Clinical:			
1. Age	1.09	1.03–1.16	0.005*
Co-morbidities:			
1. COPD	5.24	1.84–14.91	0.002*
2. PVE	3.29	1.31–8.29	0.012*
Preoperative echocardiography:			
1. LVEF below 50%	3.28	1.50–7.16	0.003*
Pre-operative laboratory parameters:			
1. SIRI on admission > 1.27	6.16	2.17–17.48	0.012*
Postoperative laboratory parameters:			
1. Platelets count	0.99	0.98–0.99	0.002*
2. Lymphocytes count	0.35	0.13–0.92	0.034*

Abbreviations: COPD- chronic pulmonary obstructive disease, PVE–peripheral vascular events in history, LVEF-left ventricle ejection fraction, SIRI–systemic inflammatory response index.

## Discussion

The main finding of our study is the predictive value of pre-operative inflammatory activity characterized by systemic inflammatory reaction index (SIRI) for long-term mortality assessment. We present the results of the multivariable analysis of clinical and laboratory pre-operative factors in patients undergoing surgical revascularization due to complex coronary disease, suggesting that inflammatory reactivity possesses a high predictive significance for long-term results. Patients requiring surgical revascularization have an individual propensity for inflammatory activation that influences their post-operative survival [17, 18]. According to our analysis those patients with more advanced inflammatory status before surgery, have higher risk of death. Therefore, we believe that this particular group of patients should undergo more scrutinous follow-up controls to improve long-term survival. Potentially, pharmacological treatment which may diminish the inflammatory activation in the atherosclerotic plaque, would be beneficial in this group of patients. Currently, statins have proved anti-inflammatory effect on the formation of atherosclerotic changes [19, 20].

Inflammatory processes activate atherosclerosis, or more accurately, its development and progression characterized by inflammatory markers and indices [21, 22]. Several previous studies [8, 23, 24] proved the relationship between inflammation, inflammatory cells activation and coronary artery disease occurrence. The degree of atherosclerotic changes in coronary arteries may be evaluated with the use of currently available diagnostic tools including coronary angiography, optical coherence tomography or cardiac magnetic resonance [25]. The measurement of inflammatory intensity influencing the atherosclerosis progression would be also beneficial. The inflammation reaction can be characterized by simple parameters obtained from the whole blood count [17, 18, 26, 27]. Those parameters, including the neutrophil to lymphocyte ratio (NLR) or monocyte to lymphocyte ratio (MLR), have predictive values for long-term mortality in off-pump patients after the surgical procedure [8]. The significance of our retrospective analysis is proposing SIRI as the pre-operative factor to predict worse late survival. We believe that searching for pre-operative factors which can reveal subgroups of patients prone to diminished long-term results is crucial. Our most valuable finding is the fact that easily available index (SIRI) may reflect higher mortality risk and therefore it is worth to include in daily practice in patients with coronary artery disease qualified for cardiac surgery.

The significance of inflammatory reactions as a possible trigger in broad spectrum of cardiovascular diseases is currently strongly underlined. The inflammatory activation presented by indexes in our study point out those patients who are more prone for complex artery disease development with secondary higher risk for worse outcomes. Previous studies revealed the relationship between inflammation and other cardiovascular risk factors and events. Szczepaniak et al. [28] presented the inflammatory link between hypertension and periodontitis. Inflammatory reaction related to nosocomial infection was described as a possible risk factor for worse presentation and outcomes after acute coronary syndrome [29]. Moreover, Fan et al. [30] identified a series of key genes closely related with inflammatory response and atrial fibrillation. The dietary intake influencing the inflammatory reactions in men that possess long-term results in increased risk for acute coronary syndromes was presented in Sut et al. [31] study. The relation between chronic inflammatory activation and a risk for left ventricular dysfunction was found in Kloch et al. [32] analysis.

The SIRI components as neutrophils, monocytes and lymphocytes play a significant role in atherosclerotic plaques formation and destabilization [33]. The initial destruction of endothelium cause monocytes adhesion and influx into intimal lawyer of the vascular wall with secondary secretion of cytokines and proteolytic enzymes [34]. The relation between hyperlipidaemia and neutrophilia [35] and monocytes activation [36] was presented in previous studies. The further modulation of monocytes/macrophages and foam cells at the site of lipids accumulation and regulated by neutrophils as these groups of cells interact in atherosclerotic plaques development [37].

The atherosclerotic plaque destabilization is of significant importance related to inflammatory cells activations. The role of innative inflammatory cells like monocytes [38] or indexes as possible markers as neutrophil to lymphocyte ratio [39] is postulated in plaques instability prediction.

Undoubtfully, both, on-pump and off-pump methods might cause an inflammatory response. The off-pump method of surgical procedures described in our analysis allowed us to minimalize the influence of post-operative inflammatory response on our results. Several studies confirmed that the off-pump surgical revascularization technique omits the risk for inflammatory activation secondary to CBP administration [40–43]. In the Mirhafez et al. [41] study serum cytokines levels in off-pump coronary surgery were lower compared with on-pump method. Despite some disadvantages, the off-pump technique still possesses a profound protective value in high-risk patients [44, 45]. The main advantage of surgical revascularization is the time-related long-term survival rate [46]. Despite its invasive nature, the surgery gives superior results and places the surgical procedure as the optimal treatment for complex coronary artery disease [47–49] especially with ulitity of arterial grafts [50, 51].

Our analysis focused on pre-operative factors that may interfere with long-term prognosis. In multivariable analysis, the diminished left ventricle ejection fraction, combined with co-morbidities such as COPD and stroke, were presented significant which is consistent with the previous reports [52, 53]. Importantly, results from previous research focused on post-operative prognostic parameters [17]. To our best knowledge, our study is the first to reveal pre-operative inflammatory activation as patients’ dependent characteristics that may interfere with post-operative results.

We noticed higher percentage of hypercholesterolemia in the survivor group. The serum LDL cholesterol fraction levels was insignificant, presenting effective therapy. However, this phenomenon was not statistically significant neither in the uni- nor in multivariable analysis. We shall point out that all patients referred for surgery reported long-lasting statin therapy, and the obtained results depended on the individual response to prescribed medication. Moreover, the results obtained for analysis were found irrelevant to hypercholesterolemia, as the target LDL serum levels were achieved suggesting more aggressive therapy in hypercholesterolemia group.

The long-term results following the off-pump coronary artery bypass grafting procedure depend on pre-operative, intraoperative, and post-operative factors [18]. Our analysis points out that among patients referred for off-pump coronary surgery, a subgroup is characterized by excessive inflammatory activity that interferes with post-operative results and long-term survival.

The present study has several limitations. It is a retrospective, single-centre study limited to less than 200 cases. However, we included all consecutive patients who met the inclusion criteria, thus we showed the real-life cohort of patients. There is a gender discrepancy in presented population, but the consecutive patients were enrolled into the analysis and the same inequality is present in clinical practise. Moreover, we only analysed patients with stable complex coronary disease. We aimed to form the most homogenic study group, therefore, acute coronary syndrome was an exclusion criterium. Patients with concomitant diseases requiring surgical intervention were not included due to already proved worse results of combined procedures [54]. We believe the broader cohort of patients, preferably including multicentre research is necessary for further investigation.

## Conclusions

The systemic inflammatory response index is a prognostic factor for worse long-term outcomes after off-pump coronary artery bypass grafting in patients with chronic coronary syndrome. A pre-operative value of SIRI above 1.27 indicates patients with higher long-term mortality risk.

## References

[1] Sousa-Uva M, Neumann FJ, Ahlsson A, et al. ESC Scientific Document Group. 2018 ESC/EACTS Guidelines on myocardial revascularization. Eur J Cardiothorac Surg. 2019; 55(1): 4–90. doi: 10.1093/ejcts/ezy289 30165632

[2] Takahashi K, Serruys PW, Fuster V, et al. SYNTAXES, FREEDOM, BEST, and PRECOMBAT trial investigators. Redevelopment and validation of the SYNTAX score II to individualise decision making between percutaneous and surgical revascularisation in patients with complex coronary artery disease: secondary analysis of the multicentre randomised controlled SYNTAXES trial with external cohort validation. Lancet. 2020;396(10260):1399–1412. doi: 10.1016/S0140-6736(20)32114-0 33038944

[3] Torregrossa G, Amabile A, Williams EE, et al. Multi-arterial and total-arterial coronary revascularization: Past, present, and future perspective. J Card Surg. 2020; 35(5): 1072–1081. doi: 10.1111/jocs.14537 32293059

[4] Zhu Y, Xian X, Wang Z et al. Research Progress on the Relationship between Atherosclerosis and Inflammation. Biomolecules. 2018; 8(3): 80–91. doi: 10.3390/biom8030080 30142970 PMC6163673

[5] Galkina E, Ley K. Immune and inflammatory mechanisms of atherosclerosis (*). Annu Rev Immunol. 2009; 27: 165–197. doi: 10.1146/annurev.immunol.021908.132620 19302038 PMC2734407

[6] Proctor MJ, McMillan DC, Horgan PG, Fletcher CD, Talwar D, Morrison DS. Systemic inflammation predicts all-cause mortality: a glasgow inflammation outcome study. PLoS One. 2015 Mar 2; 10(3): e0116206. doi: 10.1371/journal.pone.0116206 25730322 PMC4346265

[7] Gimbrone MA Jr, García-Cardeña G. Endothelial Cell Dysfunction and the Pathobiology of Atherosclerosis. Circ Res. 2016; 118(4): 620–636. doi: 10.1161/CIRCRESAHA.115.306301 26892962 PMC4762052

[8] Urbanowicz T, Olasińska-Wiśniewska A, Michalak M, et al. The Prognostic Significance of Neutrophil to Lymphocyte Ratio (NLR), Monocyte to Lymphocyte Ratio (MLR) and Platelet to Lymphocyte Ratio (PLR) on Long-Term Survival in Off-Pump Coronary Artery Bypass Grafting (OPCAB) Procedures. Biology (Basel). 2021; 11(1): 34–51. doi: 10.3390/biology11010034 35053032 PMC8772913

[9] Aranda-Michel E, Bianco V, Kilic A, et al. Mortality and Readmissions After On-Pump Versus Off-Pump Redo Coronary Artery Bypass Surgery. Cardiovasc Revasc Med. 2020; 21(7): 821–825. doi: 10.1016/j.carrev.2019.12.008 31836478

[10] Knapik P, Hirnle G, Kowalczuk-Wieteska A, O Zembala M, Pawlak S, Hrapkowicz T, et al.; Off-pump versus on-pump coronary artery surgery in octogenarians (from the KROK Registry). PLoS One. 2020; 15(9): e0238880. doi: 10.1371/journal.pone.0238880 32913359 PMC7482977

[11] David CW, John GL, John FB, et al. The Systemic Inflammatory Response to Cardiac Surgery: Implications for the Anesthesiologist. Anesthesiology 2002; 97: 215–252. doi: 10.1097/00000542-200207000-00030 12131125

[12] Wu M, Yang S, Feng X, Li C, Yu F, Dong J. Prognostic value of the postoperative neutrophil-lymphocyte ratio in solid tumors: A meta-analysis. PLoS One. 2021; 16(4): e0250091. doi: 10.1371/journal.pone.0250091 33872342 PMC8055017

[13] Peng Y, Huang W, Shi Z, et al. Positive association between systemic immune-inflammatory index and mortality of cardiogenic shock. Clin Chim Acta. 2020; 511: 97–103. doi: 10.1016/j.cca.2020.09.022 33045194

[14] Jentzer JC, Lawler PR, van Diepen S, et al. Systemic Inflammatory Response Syndrome Is Associated With Increased Mortality Across the Spectrum of Shock Severity in Cardiac Intensive Care Patients. Circ Cardiovasc Qual Outcomes. 2020; 13(12): 1033–1045. doi: 10.1161/CIRCOUTCOMES.120.006956 33280435

[15] Aydın C, Engin M. The Value of Inflammation Indexes in Predicting Patency of Saphenous Vein Grafts in Patients With Coronary Artery Bypass Graft Surgery. Cureus. 2021; 13(7): e16646–e16655. doi: 10.7759/cureus.16646 34462681 PMC8387011

[16] Dey S, Kashav R, Kohli JK, et al. Systemic Immune-Inflammation Index Predicts Poor Outcome After Elective Off-Pump CABG: A Retrospective, Single-Center Study. J Cardiothorac Vasc Anesth. 2021; 35(8): 2397–2404. doi: 10.1053/j.jvca.2020.09.092 33046365

[17] Urbanowicz T, Michalak M, Gąsecka A, et al. Postoperative Neutrophil to Lymphocyte Ratio as an Overall Mortality Midterm Prognostic Factor following OPCAB Procedures. Clin Pract. 2021; 11(3): 587–597. doi: 10.3390/clinpract11030074 34563003 PMC8482266

[18] Urbanowicz TK, Michalak M, Gąsecka A, et al. A Risk Score for Predicting Long-Term Mortality Following Off-Pump Coronary Artery Bypass Grafting. J Clin Med. 2021; 10(14): 3032–3046. doi: 10.3390/jcm10143032 34300198 PMC8305554

[19] Diamantis E, Kyriakos G, Quiles-Sanchez LV et al. The Anti-Inflammatory Effects of Statins on Coronary Artery Disease: An Updated Review of the Literature. Curr Cardiol Rev. 2017; 13(3): 209–216. doi: 10.2174/1573403X13666170426104611 28462692 PMC5633715

[20] van der Meij E, Koning GG, Vriens PW, Peeters MF, Meijer CA, Kortekaas KE, et al. A clinical evaluation of statin pleiotropy: statins selectively and dose-dependently reduce vascular inflammation. PLoS One. 2013; 8(1): e53882. doi: 10.1371/journal.pone.0053882 23349755 PMC3551939

[21] Kocyigit D, Gurses KM, Tokgozoglu L. Anti-inflammatory therapy in atherosclerosis. Front Biosci (Landmark Ed). 2020; 25: 242–269. doi: 10.2741/4805 31585888

[22] Heeneman S, Donners MM, Bai L, et al. Drug-induced immunomodulation to affect the development and progression of atherosclerosis: a new opportunity? Expert Rev Cardiovasc Ther. 2007; 5(2): 345–364. doi: 10.1586/14779072.5.2.345 17338677

[23] Barrett TJ. Macrophages in Atherosclerosis Regression. Arterioscler Thromb Vasc Biol. 2020; 40(1): 20–33. doi: 10.1161/ATVBAHA.119.312802 31722535 PMC6946104

[24] Boland J, Long C. Update on the Inflammatory Hypothesis of Coronary Artery Disease. Curr Cardiol Rep. 2021; 23(2): 6–12. doi: 10.1007/s11886-020-01439-2 33409720

[25] Iwańczyk S, Skorupski WJ, Kępski S et al. Thromboembolic or atherosclerotic? Optical coherence tomography in determining the cause of myocardial infarction with ST-segment elevation. Kardiol Pol. 2020; 78(10): 1045–1046. doi: 10.33963/KP.15499 Epub 2020 Jul 8. . 32640773

[26] Weymann A, Ali-Hasan-Al-Saegh S, Popov AF et al. Haematological indices as predictors of atrial fibrillation following isolated coronary artery bypass grafting, valvular surgery, or combined procedures: a systematic review with meta-analysis. Kardiol Pol. 2018; 76(1): 107–118. doi: 10.5603/KP.a2017.0179 . 28980298

[27] Sari I, Sunbul M, Mammadov C et al. Relation of neutrophil-to-lymphocyte and platelet-to-lymphocyte ratio with coronary artery disease severity in patients undergoing coronary angiography. Kardiol Pol. 2015; 73(12): 1310–1316. doi: 10.5603/KP.a2015.0098 Epub 2015 May 19. . 25987404

[28] Szczepaniak P, Mikołajczyk TP, Cześnikiewicz-Guzik M, et al. Periodontitis as an inflammatory trigger in hypertension: From basic immunology to clinical implications. Kardiol Pol. 2021; 79(11): 1206–1214. doi: 10.33963/KP.a2021.0161 34847238

[29] Santos M, Oliveira M, Vieira S, et al. Predictors and mid-term outcomes of nosocomial infection in ST-elevation myocardial infarction patients treated by primary angioplasty. Kardiol Pol. 2021; 79(9): 988–994. doi: 10.33963/KP.a2021.0058 34231873

[30] Fan G, Wei J. Identification of potential novel biomarkers and therapeutic targets involved in human atrial fibrillation based on bioinformatics analysis. Kardiol Pol. 2020; 78(7–8): 694–702. doi: 10.33963/KP.15339 32383373

[31] Sut A, Chiżyński K, Różalski M, et al. Dietary intake of omega fatty acids and polyphenols and its relationship with the levels of inflammatory markers in men with chronic coronary syndrome after percutaneous coronary intervention. Kardiol Pol. 2020; 78(2): 117–123. doi: 10.33963/KP.15078 31790083

[32] Kloch M, Stolarz-Skrzypek K, Olszanecka A, et al. Inflammatory markers and left ventricular diastolic dysfunction in a family-based population study. Kardiol Pol. 2019; 77(1): 33–39. doi: 10.5603/KP.a2018.0214 30406940

[33] Mehu M, Narasimhulu CA, Singla DK. Inflammatory Cells in Atherosclerosis. Antioxidants (Basel). 2022; 11(2): 233–251. doi: 10.3390/antiox11020233 35204116 PMC8868126

[34] Gautam N, Olofsson AM, Herwald H, Iversen LF, Lundgren-Akerlund E, Hedqvist P, et al. Heparin-binding protein (HBP/CAP37): a missing link in neutrophil-evoked alteration of vascular permeability. Nat Med. 2001; 7(10): 1123–1127. doi: 10.1038/nm1001-1123 11590435

[35] Drechsler M, Megens RT, van Zandvoort M, Weber C, Soehnlein O. Hyperlipidemia-triggered neutrophilia promotes early atherosclerosis. Circulation. 2010; 122(18): 1837–45. doi: 10.1161/CIRCULATIONAHA.110.961714 20956207

[36] Moroni F, Ammirati E, Norata GD, Magnoni M, Camici PG. The Role of Monocytes and Macrophages in Human Atherosclerosis, Plaque Neoangiogenesis, and Atherothrombosis. Mediators Inflamm. 2019; 2019: 7434376. doi: 10.1155/2019/7434376 31089324 PMC6476044

[37] Prame Kumar K, Nicholls AJ, Wong CHY. Partners in crime: neutrophils and monocytes/macrophages in inflammation and disease. Cell Tissue Res. 2018; 371(3): 551–565. doi: 10.1007/s00441-017-2753-2 29387942 PMC5820413

[38] Vinci R, Pedicino D, Bonanni A, D’Aiello A, Severino A, Pisano E, et al. A Novel Monocyte Subset as a Unique Signature of Atherosclerotic Plaque Rupture. Front Cell Dev Biol. 2021; 9: 753223. doi: 10.3389/fcell.2021.753223 34712669 PMC8545820

[39] Shimonaga K, Matsushige T, Takahashi H, Hashimoto Y, Yoshiyama M, Ono C, et al. Peptidylarginine Deiminase 4 as a Possible Biomarker of Plaque Instability in Carotid Artery Stenosis. J Stroke Cerebrovasc Dis. 2021; 30(7): 105816. doi: 10.1016/j.jstrokecerebrovasdis.2021.105816 33906071

[40] Hadem J, Rossnick R, Hesse B, et al. Endothelial dysfunction following coronary artery bypass grafting: Influence of patient and procedural factors. Herz. 2020; 45(1): 86–94. doi: 10.1007/s00059-018-4708-0 29774399

[41] Mirhafez SR, Khadem SH, Sahebkar A et al. Comparative effects of on-pump versus off-pump coronary artery bypass grafting surgery on serum cytokine and chemokine levels. IUBMB Life. 2021; 73(12): 1423–1431. doi: 10.1002/iub.2566 Epub 2021 Oct 15. . 34601812

[42] Møller CH, Steinbrüchel DA. Off-pump versus on-pump coronary artery bypass grafting. Curr Cardiol Rep. 2014; 16(3): 455–465. doi: 10.1007/s11886-013-0455-2 . 24482012

[43] Merkle J, Sunny J, Ehlscheid L, Sabashnikov A, Weber C, Eghbalzadeh K, et al. Early and long-term outcomes of coronary artery bypass surgery with and without use of heart-lung machine and with special respect to renal function—A retrospective study. PLoS One. 2019; 14(10): e0223806. doi: 10.1371/journal.pone.0223806 31600308 PMC6786630

[44] Sondekoppam RV, Arellano R, Ganapathy S et al. Pain and inflammatory response following off-pump coronary artery bypass grafting. Curr Opin Anaesthesiol. 2014; 27(1):v106–115. doi: 10.1097/ACO.0000000000000036 . 24322210

[45] Guida GA, Chivasso P, Fudulu D, et al. Off-pump coronary artery bypass grafting in high-risk patients: a review. J Thorac Dis. 2016; 8(Suppl 10): S795–S798. doi: 10.21037/jtd.2016.10.107 27942397 PMC5124583

[46] Dominici C, Salsano A, Nenna A, et al. On-pump beating-heart coronary artery bypass grafting in high-risk patients: A systematic review and meta-analysis. J Card Surg. 2020; 35(8): 1958–1978. doi: 10.1111/jocs.14780 32643847

[47] Stone GW, Kappetein AP, Sabik JF, et al. EXCEL Trial Investigators. Five-Year Outcomes after PCI or CABG for Left Main Coronary Disease. N Engl J Med. 2019; 381(19): 1820–1830. doi: 10.1056/NEJMoa1909406 31562798

[48] De Palo M, Quagliara T, Dachille et al. Trials Comparing Percutaneous And Surgical Myocardial Revascularization: A Review. Rev Recent Clin Trials. 2019; 14(2): 95–105. doi: 10.2174/1574887114666190201102353 30706789

[49] Fertouk M, Gordon A, Pevni D, Ziv-Baran T, Sela O, Mohr R, et al. Early and late outcomes of single versus bilateral internal thoracic artery revascularization for patients in critical condition. PLoS One. 2021; 16(8): e0255740. doi: 10.1371/journal.pone.0255740 34352035 PMC8341519

[50] Torregrossa G, Amabile A, Fonceva A, Hosseinian L, Williams EE, Balkhy HH, et al. Outcomes in Complete Arterial Coronary Revascularization. J Cardiothorac Vasc Anesth. 2020; 34(12): 3444–3448. doi: 10.1053/j.jvca.2020.03.017 32359710

[51] Huckaby LV, Sultan I, Ferdinand FD et al. Matched Analysis of Surgical Versus Percutaneous Revascularization for Left Main Coronary Disease. Ann Thorac Surg. 2022; 113(3): 800–807. doi: 10.1016/j.athoracsur.2021.04.043 33930354

[52] Sergeant P, Wouters P, Meyns B, et al. OPCAB versus early mortality and morbidity: an issue between clinical relevance and statistical significance, European Journal of Cardio-Thoracic Surgery. 2004; 25(5): 779–785. doi: 10.1016/j.ejcts.2004.02.013 15082282

[53] Shinjo D, Fushimi K. Preoperative factors affecting cost and length of stay for isolated off-pump coronary artery bypass grafting: hierarchical linear model analysis. BMJ Open 2015; 5: e008750–e008759. doi: 10.1136/bmjopen-2015-008750 26576810 PMC4654398

[54] Perek B, Misterski M, Stachowiak W, et al. The impact of coronary artery disease severity on late survival after combined aortic valve replacement and coronary artery bypass grafting—experience of a single cardiac surgery center. Kardiochir Torakochirurgia Pol. 2014; 11(4): 361–366. doi: 10.5114/kitp.2014.47333 26336450 PMC4349042

