# Supplementary material for: Pre-operative systemic inflammatory response index influences long-term survival rate in off-pump surgical revascularization
Source: PLoS One. 2022 Dec 15;17(12):e0276138. doi: 10.1371/journal.pone.0276138 (PMC9754600; doi:10.1371/journal.pone.0276138)
Supplement: Attachment — Submitted filename: Reviewer1 answers.docx [file pone.0276138.s001.docx]

Poznań, 7.6.2022

Dear Reviewer,

I would like to thank you for your valuable comments.

The answers are presented below.

If you have further suggestions, I would be more than happy to answer.

Kind regards

Tomasz Urbanowicz

On behalf of all co-authors

Reviewer #1: PONE-D-22-08151

Reviewer comment - The present manuscript aim to explore the possible predictive role of preoperative inflammatory indexes after the off-pump coronary artery bypass grafting technique on long-term survival. The study indicate that preoperative inflammatory activation, evaluated by systemic inflammatory reaction index (SIRI) possesses a prognostic value for complex coronary artery disease patients. A SIRI value above 1.27 indicates a worse late prognosis after off-pump coronary artery bypass.

The authors took up an interesting topic of research, but I have some comments to address them:

1. Materials and methods. A) The authors stated to include additional laboratory parameters other than blood count. Which ones?

- We included troponin level. The information is included in the Methods section

Did they evaluated other inflammatory mediators such as C-reactive protein?

- Pre-procedural CRP was not routinely measured except from patients with signs of infection – those subjects were however excluded from the study group. We did not evaluated CRP levels postoperatively and did not correlated them with other markers as this marker was not routinely collected after surgery.

B) the definition of PVE and PAD is a bit confusing both in the test and in the tables. Sometimes the authors speak of PVE and others of stroke… Sometimes they use the abbreviations other the full name (Table 1. Point 4 and 5).

Thank you for this valuable comment – we unified the definitions.

2. Results section: data is not well presented. I believe it is necessary to show a table with all the preoperative demographic and clinical characteristics of the study population.

Table 1 presents pre-operative characteristics of the study group
The laboratory parameters, significantly different between the surviving and non-surviving groups, can be presented as **box-plot images**, instead of Table 2.

Thank you for this valuable comments – we added box-plot images.
Furthermore, the univariate table (Table 3) must contain all the variables that have been analyzed to better understand which enter the multivariable model.

Thank you for this valuable comments – we added all variables in the univariate table .

I also would like to ask the authors to explain why they think hypercholesterolemia is higher in survivors than in non-survivors.
Did they also consider the different drug treatments?

We noticed higher percentage of hypercholesterolemia in the survivor group. However, this phenomenon was not statistically significant neither in the uni- nor in multivariable analysis. We shall point out that all patients referred for surgery reported long-lasting statin therapy, and the obtained results depended on the individual response to prescribed medication. We did not evaluated target serum levels of cholesterol’ fraction. Moreover, the results obtained for analysis were relevant for the time of surgery and the initial lipid profile characteristics was not available for the analysis. Therefore, the cholesterol level changes, and achievement of treatment goals were not evaluated and, in our opinion, should not be treated as a prognostic factor.
